# Prevalence of and factors associated with burnout in midwifery: A scoping review

**DOI:** 10.18332/ejm/115983

**Published:** 2020-02-11

**Authors:** Rawel Sidhu, Bowen Su, Kate R. Shapiro, Kathrin Stoll

**Affiliations:** 1 Vancouver Fraser Medical Program, Faculty of Medicine, University of British Columbia, Vancouver, Canada; 2 Division of Midwifery, Department of Family Practice, University of British Columbia, Vancouver, Canada

**Keywords:** stress, midwives, burnout, scoping review

## Abstract

**INTRODUCTION:**

Midwifery care meets the triple aims of health system improvement, i.e. good health outcomes, high client satisfaction, and low per capita costs. Scaling up access to midwifery care is a global priority yet the growth and sustainability of the profession is threatened by high levels of burnout and attrition. This scoping review provides a comprehensive review of the existing literature on burnout in midwifery, with a focus on prevalence, associated factors and potential solutions.

**METHODS:**

Four electronic databases were searched to locate relevant literature up to July 2019. A total of 1034 articles were identified and reduced to 27 articles that met inclusion criteria. We summarize sample sizes, settings, study designs, burnout measures, prevalence of burnout, associated factors and potential solutions, and recommendations.

**RESULTS:**

Prevalence of burnout was highest among Australian, Western Canadian and Senegalese midwives and lowest among Dutch and Norwegian midwives. Midwives working in caseload/continuity models reported significantly lower burnout compared to midwives working in other models. We identified 26 organizational and personal factors that were significantly associated with burnout, such as high workload, exposure to traumatic events, and fewer years in practices. Organizational support to improve work-life balance and emotional well-being, as well as more continuing education to raise awareness about burnout and how to cope with it, emerged as common strategies to prevent and address burnout.

**CONCLUSIONS:**

Burnout is a serious and complex occupational phenomenon. More qualitative research is needed in this area, to better understand the lived experience of burnout.

## INTRODUCTION

There is a large and growing body of literature documenting the positive impact midwives have on the healthcare system. Midwifery care is linked to fewer birth complications, reducing the need for obstetrical intervention, alleviating provider shortages in underserved communities, and making more efficient use of health care funding^1^. While there is mounting evidence that midwifery care meets the triple aims of health system improvement (good outcomes, high patient satisfaction/good experiences of care, and low per capita costs) the growth and sustainability of the midwifery profession in some countries and regions is threatened by high levels of burnout and attrition^2,3^. For example, in a Danish study of burnout among 15 professional groups, midwives reported the highest personal and work-related burnout scores^4^. Recognizing the experiences of health care providers and the effect of burnout on quality of care, the triple aims were expanded to include a fourth aim: improving the work lives and well-being of health professionals^5^. This scoping review focuses on provider experiences, specifically prevalence, associated factors and potential solutions to burnout.

Burnout – defined as chronic occupational-stress resulting in a loss of energy, dissociation from work, depersonalization, and emotional exhaustion – has received increasing attention in the literature^3,6^. Central to this conversation has been the association observed between high burnout and poorer quality of care, low job satisfaction, and employee resignation^3,6,7^. Midwives, in particular, are vulnerable to experiencing burnout for several reasons: they report having fewer resources than their peers in other health care professions, their work often extends past their contracted time forcing them to miss breaks, and they feel inadequately compensated for the work they perform^3,8,9^. As well, several individual factors such as having a high level of empathetic identification with women and struggling to process poor maternal-fetal outcomes have also been indicated as factors that contribute to a midwife’s vulnerability to burnout^10,11^.

Despite the well-recognized problem of burnout in midwifery, there are comparably few studies that have systematically examined the prevalence of and factors that are associated with burnout in the midwifery profession. The research question guiding this review was: ‘How common is burnout in midwifery, which factors are associated with burnout and which solutions or recommendations have been published, to address this issue?’.

## METHODS

We adopted a scoping review methodology as outlined by Arksey and O’Malley^12^. A scoping review is a type of descriptive literature review that maps key concepts in a certain area of the literature. Unlike a systematic review, which addresses a well-defined study question from a narrow range of appropriately designed studies, a scoping review is best suited for broadly defined research aims for when the literature is widely heterogenous in study design, theoretical framework, or outcomes measured – as was found to be the case for burnout in midwifery. Accordingly, a five-step approach for conducting a scoping review was used: 1) Identifying a research question (see Introduction); 2) Identifying relevant studies; 3) Selecting relevant studies; 4) Charting the data; and 5) Collating, summarizing, and reporting the results.

### Identifying relevant studies

Several electronic databases were searched to identify relevant studies up to July 2019: Medline, CINAHL, PsychINFO, and PubMed. Keywords chosen in the search included ‘midwife’ (and its variations, e.g. midwifery, midwives), in combination with burnout-related terms including ‘burnout,’ ‘exhaustion,’ and ‘compassion fatigue.’ The search strategies were tailored for each of the databases’ thesaurus terms and field headings. A total of 1034 articles were identified (Medline=219; CINAHL=203; PsychINFO=151; PubMed=461). Eliminating all non-English articles and removing duplicates reduced this number to 598 articles eligible for title and abstract review. From these, 92 articles were deemed to be relevant and underwent closer review. Articles were evaluated on the following inclusion criteria: 1) Article must be on practicing midwives (e.g. studies involving nurse-midwives were included, but articles on student midwives or retired midwives were excluded); 2) Article must report on burnout among midwives; if other healthcare providers were included, results must be stratified, so that midwifery-specific results can be extracted; 3) Article must identify associated factor(s); and 4) Article must be written in English and have full-text available. A total of 27 articles met these criteria (Table 1).

**Table 1:** Relevant studies

*Article title*	*N*	*Country*	*Study design*	*Burnout measure (if used)*	*Prevalence of burnout*	*Factors associated with burnout*	*Recommendations for addressing burnout*
A survey of burnout and intentions to leave the profession among Western Canadian midwives^3^	158 midwives from Western Canada	Canada	Quantitative cross-sectional survey	CBI^1^	Severe or moderate burnout 43.4%, personal 74.9%, work-related 42.5%, client-related 20.3% Mean/median burnout scores: total 45.0/47.4, personal 60.4/62.5, work-related 6.8/46.4, client-related 8.5/29.2	–Planning to leave the profession –Having young children –Fewer days off –Having symptoms of anxiety, depression and stress –Reporting more negative/challenging practice environments –Not feeling valued by team members	–Part-time work options –Support for sick days/vacation coverage –More pay per course of care –More off-call career opportunities –Initiatives to reduce bullying and inter-professional conflict –Creating practice environments where midwives feel safe and self-care is valued
Burnout among Norwegian midwives and the contribution of personal and work-related factors: A cross-sectional study^7^	598 Norwegian midwives	Norway	Quantitative cross-sectional survey	CBI	Personal burnout 20.1%, work-related 19.1 %, client-related 4.2 %	–Sick leave within the last 3 months –Being single –Working in outpatient care –Experiencing recent reorganization at work –Being younger (<60 years old)	N/A
Factors that may influence midwives work-related stress and burnout^11^	56 registered midwives	Australia	Quantitative cross-sectional survey	MBI^2^	High to moderate emotional exhaustion 60.7%, high to moderate depersonalization 30.3%, low personal accomplishment 30.3%	–Less work experience –Night shifts or mixed night/day shifts –More clients with complex needs –Low exercise	–More organizational support and exercise
Is caseload midwifery a healthy work-form? - A survey of burnout among midwives in Denmark^13^	50 midwives at one hospital	Denmark	Quantitative cross-sectional survey	CBI	Personal burnout mean score and SD 37.6±16.2, work-related 35.0±15.7, client-related 26.5±16.4	- Midwives working in caseload model reported significantly lower scores on all three domains of burnout, compared to noncaseload midwives	-Further research on how continuity of care models impact on emotional well-being
Prevalence of burnout, depression, anxiety and stress among Australian midwives: A cross-sectional study^14^	1037 midwives	Australia	Quantitative cross-sectional survey	CBI	Personal burnout mean score and SD 55.9±18.1, work-related 48.4±17.4, client related 25.6±18.3	–Having symptoms of depression, anxiety, and/or stress	N/A
The emotional wellbeing of New Zealand midwives: Comparing responses for midwives in caseloading and shift work settings^15^	1073 midwives responded with 44% (n=473) self-employed, 42% (n=452) employed and 14% (n=148) both selfemployed and employed	New Zealand	Quantitative cross-sectional survey	CBI	Self-employed: personal burnout mean score and SD 52.49±16.71, work-related 39.67±18.21, client-related 23.85±20.30 Employed: personal burnout mean score and SD 53.93±18.42, work-related 42.81±19.82, client-related 22.93±19.87 Employed and self-employed: personal burnout mean score and SD 49.17±16.63, work-related 37.69±16.49, client-related 20.0±15.72	–Employed midwives reported more burnout than self-employed midwives and also reported lower levels of autonomy, empowerment and professional recognition. –Burnout was higher among midwives who also reported resource inadequacy, lack of development opportunities and poor management quality –Younger age –More hours worked per week –Poor interprofessional relationships –More years working as midwife	–Having enough midwives to provide quality care, i.e. change to midwifery staffing standards at hospitals –Presence of supportive midwifery manager
The effects of midwives’ job satisfaction on burnout, intention to quit and turnover: a longitudinal study in Senegal^16^	226 midwives	Senegal	Quantitative longitudinal survey study (2 years)	MBI	55% of respondents identified as being burned out High emotional exhaustion 80%, depersonalization 57.8%, low personal accomplishment 12.4%	–Dissatisfaction with pay –Low task satisfaction	–Continuing education and professional opportunities for midwives, to avoid attrition
Comparing caseload and non-caseload midwives’ burnout levels and professional attitudes: A national, cross-sectional survey of Australian midwives working in the public maternity system^17^	542 midwives across 111 hospitals from all Australian states and one of the territories	Australia	Quantitative cross-sectional survey	CBI	Caseload midwives: personal burnout mean score and SD 39.8±18.8, work-related 36.6±19.9, client-related 17.9±18.7 Non-caseload midwives: personal burnout mean score and SD 44.8.8±20.4, work-related 45.9±20.6, client-related 18.9.9±17.4	–Non-caseload midwives had significantly higher personal and work burnout scores, compared to case loading midwives	–Practicing in a caseload model may reduce burnout
Comparing satisfaction and burnout between caseload and standard care midwives: findings from two cross-sectional surveys conducted in Victoria, Australia^18^	20 caseload midwives and 130 standard care midwives responded at baseline; 22 caseload midwives and 133 standard care midwives responded at two years follow-up	Australia	Two quantitative cross-sectional surveys, one administered at commencement of caseload midwifery model and one survey two years later	CBI	Caseload midwives: personal burnout mean score and SD 44.2±21.2, work-related 41.1±21.6, client-related 12.3±9.6 Non-caseload midwives: personal burnout mean score and SD 50.1±17.5, work-related 45.1±18.5, client-related 22.4.3±18.0	–Non-caseload model of midwifery was linked to significantly higher personal, work and client related burnout at two years	–Practicing in a caseload model may reduce burnout
Level of burnout in a small population of Australian midwives^19^	58 midwives	Australia	Quantitative cross-sectional survey	CBI	Nearly 30% reported moderate to high levels of burnout Personal burnout 57%, work-related 57%, client-related 9%	–Younger midwives (<35 years old) reported more work and personal burnout and older midwives more client burnout Less work experience Lower pay	–Additional education and support, to build competence and confidence for midwives to work to their full scope –Clinical mentorship and reorganizing models of maternity care to increase work satisfaction and autonomy and strengthen relationships between midwives and women
Personal, professional and workplace factors that contribute to burnout in Australian midwives^20^	990 midwives	Australia	Quantitative cross-sectional survey	CBI	High or moderate burnout: personal 64.9%, work-related 43.8%, client-related 10.4%	–No children –Non-caseload midwifery care –Not working in regional areas Lack of satisfaction with work- life balance Having been registered for 5-10 years	–Family-friendly work environments that facilitate work-life balance –Opportunities to work in caseload model
The emotional and professional wellbeing of Australian midwives: A comparison between those providing continuity of midwifery care and those not providing continuity^21^	862 midwives working in continuity care (n=214) and those not working in continuity care (n=648)	Australia	Quantitative cross-sectional survey	CBI	Continuity care: median personal burnout score 50, work-related 35.7, client-related 8.3 Non-continuity care: median personal burnout score: 58.3, work-related 46.4, client-related 16.7	–Midwives working in non-continuity of care models had significantly lower scores on all burnout subscales	–Increase availability of continuity models
Exposure to traumatic perinatal experiences and post-traumatic stress symptoms in midwives: prevalence and association with burnout^22^	421 midwives with exposure to traumatic perinatal experiences	UK	Quantitative cross-sectional survey	MBI	Emotional exhaustion mean score and SD 23.8±11.5, depersonalization 3.8±4.1, personal accomplishment 38.9±5.9	–33% of respondents were symptomatic for clinical post-traumatic stress disorder –Symptoms of post-traumatic stress were associated with burnout	–Support following traumatic perinatal events –Provide intervention for those experiencing symptoms of post-traumatic stress
Occupational burnout and work factors in community and hospital midwives: A survey analysis^23^	128 midwives working at one Hospital Trust in England	UK	Quantitative cross-sectional survey	MBI	Emotional exhaustion mean score and SD 32.9±9.70, depersonalization 9.1±4.35, personal achievement 45.8±5.97	–Low occupational autonomy –More working hours – Lack of satisfaction with organizational support for work- life balance	–Organizational support for work-life balance of midwives –Ways of practicing that are linked to higher occupational autonomy, such as community midwifery
Burnout, Psychological Symptoms, and Secondary Traumatic Stress Among Midwives Working on Perinatal Wards: A Cross-Cultural Study Between Japan and Switzerland^24^	170 midwives (51 from Japan and 119 from Switzerland)	Japan and Switzerland	Quantitative cross-sectional survey	MBI	Japanese midwives: emotional exhaustion mean score and SD 20.1±9.9, depersonalization 3.2±3.7, personal accomplishment 29.7±9.5 Swiss midwives: emotional exhaustion mean score and SD 20.7±8.7, depersonalization 4.8±3.8, personal accomplishment 32.9±4.8	–Being married (Japanese midwives)	N/A
Professional quality of life of Japanese nurses/midwives providing abortion/childbirth care^25^	255 nurses and midwives working in abortion and childbirth services (86 midwives and 169 nurses)	Japan	Quantitative cross-sectional survey	ProQOL^3^ scale, which includes a burnout subscale	Mean burnout score and SD among midwives 27.0±4.9	–Burnout scores were higher among midwives and nurses who were involved in more abortions and among those who had negative emotions about providing abortion care, such as thinking that the aborted fetus deserved to live and inability to refuse involvement in abortion care	–Increase awareness about the significant distress related to abortion care among midwives and nurses –Support care providers to acquire coping skills
Psychosocial health and well-being among obstetricians and midwives involved in traumatic childbirth^26^	944 midwives and 293 obstetricians	Denmark	Quantitative cross-sectional survey	COPSOQ^4^ which includes a burnout subscale	Burnout mean score among midwives 35 (compared to 23 for obstetricians)	Following a traumatic event, female midwives reported significantly higher burnout, sleep disorders and somatic stress, compared to female obstetricians 85% of participants had experienced a traumatic childbirth	Good support after traumatic events is important for the psycho-social health and well-being of midwives and obstetricians
Burnout in Swedish midwives^27^	475 midwives	Sweden	Quantitative cross-sectional survey	CBI	High personal burnout 39.5% mean 43.0, work-related ~15% mean 33.9, client-related ~15% mean 30.4	–Age <40 years –Work experience <10 years –Conflict with team members –Lack of resources –Not having children –Worries about own health or future	N/A
Swedish midwives’ perception of their practice environment – A cross sectional study^28^	475 midwives	Sweden	Quantitative cross-sectional survey	CBI	Reported personal burnout: 183 midwives, work-related 72 midwives, client-related 68 midwives	–Midwives with burnout assessed their work environment more negatively, i.e. they scored lower on subscales measuring: –Leadership and manager related support and ability –Staffing and resources –Collegial midwife/doctor relationship	–More focus on establishing healthy work environments where midwives feel valued –Reorganizing services so midwives can practice to their full scope and provide continuity of care
‘Burnout’ among Dutch midwives^29^	200 Dutch community midwives completed	Netherlands	Two surveys and diary entries over 3-week period (to record working hours and activities)	MBI	Emotional exhaustion mean score and SD 19.9±8.2, depersonalization 6.4±3.7, personal accomplishment 33.4±4.1	–Attendance more hospital compared to home births –Lack of social support –Passive coping –Less work experience More work hours per week were linked to higher personal accomplishment	–Increase personal and work resources
Burnout experienced by nurse midwives^30^	98 nurse-midwives	USA	Quantitative cross-sectional survey	MBI	Majority of respondents experienced low levels of burnout Moderate to high emotional exhaustion: 41.8%, moderate to high personal accomplishment 32.6%, moderate to high depersonalization 26.6%	–Marital status (divorced) –Younger age –Number of children –Difficulties finding childcare –Less work experience –Low peer and consumer support –Higher proportion of clients on welfare –More deliveries or higher workload –Less pay	–More support for midwives who are serving clients with low SES –Targeted support for midwives at increased risk for burnout, such as young, newly employed midwives, who have children –More education, to help new graduate choose sites specific to their needs
Job burnout and its relation with personality traits among the midwives working in Isfahan, Iran^31^	193 midwives	Iran	Quantitative cross-sectional survey	MBI	Moderate to high emotional exhaustion 41.9%, moderate to high depersonalization 34.2%, low personal accomplishment 18.7%	–Being younger	–Younger midwives need targeted support –Educational workshops to prevent and address burnout
Mental Health Symptoms and Work-Related Stressors in Hospital Midwives and NICU Nurses: A Mixed Methods Study^32^	122 midwives and 91 NICU nurses at two Swiss university hospitals	Switzerland	Cross-sectional survey, including quantitative measures and one qualitative question in an online survey (‘Please describe briefly work- related stressors you have encountered at work in the past year’)	MBI	Midwives only: emotional exhaustion mean score and SD 20.7±8.7, depersonalization 4.8±3.8, personal achievement 32.9±4.1 High or moderate burnout: emotional exhaustion 64.7%, depersonalization 37.0%, low personal achievement 56.3%	Midwives listed maternal death and neonatal resuscitations as examples of traumatic events at work and managing patients with complex social and psychological needs as occupational stressors	Continued professional education for midwives about coping with traumatic events, e.g. CORES C: Counselling services by a professional counsellor O: Open communication through debriefing sessions following a traumatic event R: Respite care by taking time off following a traumatic event E: Education and training to help midwives cope with traumatic stress S: Support from peers
Professional burnout and social support in the workplace among hospice nurses and midwives in Poland^33^	59 midwives and 58 hospice nurses	Poland	Quantitative cross-sectional questionnaire survey	MBI	Emotional exhaustion mean score and SD 23.59±11.03, personal accomplishment 21.15±11.10, depersonalization 7.10±5.74	–Lower support from superiors and peers was correlated with higher burnout	Interventions to address burnout in midwives should focus on improving support from supervising midwives
Work-related stress, burnout and job satisfaction in Turkish midwives^34^	325 midwives	Turkey	Quantitative cross-sectional survey	MBI	Emotional exhaustion means score and SD 13.9±6.9, depersonalization 3.4±4.0, personal accomplishment 20.0±3.9	–Low work satisfaction –Work-related strain –Negative opinion about profession from clients or other midwives – Fewer than 10 years in profession	–Continuing education about how to cope with stress
Exposure to traumatic events at work, post-traumatic symptoms and professional quality of life among midwives^37^	93 midwives	Israel	Quantitative cross-sectional survey and brief descriptions of traumatic events	ProQOL scale, which includes three subscales: Compassion satisfaction, burnout and compassion fatigue/secondary trauma symptoms	On the burnout subscale 97.8% scored in the low range, while 2.2% scored in the mid-range	–Seniority (years in profession) –PTSD symptoms	–Compassion training for midwives and medical staff –Regular assessments of Professional Quality of Life, to identify midwives who need additional training and support –More research into coping mechanisms that midwives use –More research into preparing and caring for midwives who experience traumatic perinatal events
Professional Quality of Life and Associated Factors Among Ugandan Midwives Working in Mubende and Mityana Rural Districts^38^	224 midwives working in two rural districts of Uganda	Uganda	Quantitative cross-sectional survey	ProQOL	Mean burnout score and SD 36.9±6.22	–Lower education – Non-midwifery work activities –Poor physical well-being in previous year –Being married	–More support with managing stress –Counselling and debriefing after traumatic events

1 CBI: Copenhagen Burnout Inventory.

2 MBI: Maslach Burnout Inventory.

3 ProQOL: Professional Quality of Life scale.

4 COPSOQ: Copenhagen Psychosocial Questionnaire.

SD: standard deviation.

**Figure 1:**
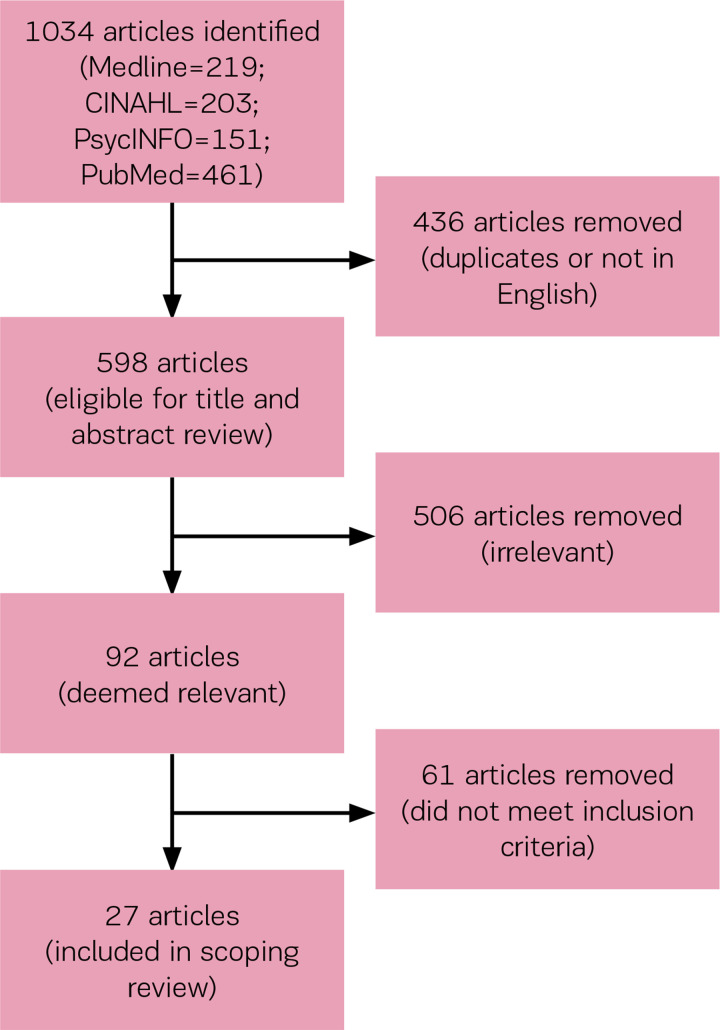
Search strategy flowchart

### Charting the data

Authors RS and BS collaborated extensively via a shared spreadsheet to review inclusion criteria and select articles, with any and all differences being settled through discussion or input from the supervising author, KS. Articles meeting the inclusion criteria were reviewed and data were extracted and charted pertaining to study setting, study design, burnout measures, study results, factors associated with burnout, and recommendations to address burnout. Factors that were significantly linked to burnout in quantitative studies were independently extracted by the first two authors and are summarized in Table 2. The supervising author reviewed all data points reported in Tables 1 and 2.

**Table 2:** Factors associated with burnout

*Factors*	*Supporting Studies*
Insufficient organizational support/distress related to organizational structure /poor or stressful work	3, 7, 15, 27, 28, 30, 33
environment/poor perceptions of practice environment	
Non case-load/non-continuity models of care (such as hospital shift work)	13, 15, 17, 18, 20, 21
Less work experience in maternity care	11, 19, 27, 29, 30, 34
Younger age	7, 15, 19, 27, 30, 31
High workload/number of work hours/fewer days off work	3, 15, 23, 27, 30
Trauma/stress experienced at work/post-traumatic stress symptoms	22, 25, 34, 37
Interpersonal conflict with colleagues/low recognition of midwives	15, 27, 28, 34
Low job/task satisfaction/non-midwifery work tasks	34, 37, 38
Lack of support from family or colleagues	13, 30, 33
Low pay	19, 30, 37
Not having children	14, 20, 27
Having (young) children/number of children	3, 30
Being single/unmarried/divorced	7, 30
Low job autonomy	23, 25
Serving clients with complex psycho-social needs	11, 30
Lack of work-life balance/lack of organizational support for work-life balance	23, 29
Seniority/more years in profession	15, 37
Being married	24, 38
Poor self-rated physical health/worries about health	27, 38
Depression/anxiety/stress	3, 14
Low/less exercise	11
Working night shifts	11
Lower percentage of home births attended	29
Passive coping style	29
Less education	38
Lack of (career) development opportunities	15

## RESULTS

Samples sizes across the 27 included studies varied, from a small survey study of 50 Danish midwives^13^, to over 1000 midwives in studies from Australia and New Zealand^14,15^. There was less variation with the study designs: all included studies used surveys to collect data; in one study two surveys were administered over a two-year period^16^.

With respect to study location, the 27 studies spanned 17 countries. Seven studies were set in Australia^11,14,17-21^, and two studies were from each of the following countries: United Kingdom^22,23^, Japan^24,25^, Denmark^13,26^, and Sweden^27,28^. See Table 1 for more details on study settings.

### Measuring burnout

The most commonly used measures to assess burnout were the Maslach Burnout Inventory (MBI), utilized in 11 of the studies^11,16,22-24,29-34^, and the Copenhagen Burnout Inventory (CBI), which was used in 12 of the 27 studies^3,7,13-15,17-21,27,28^. See Table 1 for a full list of instruments that were used to assess burnout.

The Maslach Burnout Inventory includes 22 items that measure burnout along three dimensions: emotional exhaustion, depersonalization, and reduced personal accomplishment. Each item or statement is assessed on a 7-point Likert scale. The 9 items on the emotional exhaustion subscale measure feelings of being emotionally drained and exhausted by work. The 5 items on the depersonalization subscale assess the degree to which people are impersonal in their treatment of clients/patients^35^. Personal accomplishment is measured with 8 items that assess feelings of competence and achievement with respect to work. Scores on the MBI subscales are always reported separately, whereas the CBI has both a full-scale score and three subscale scores: personal burnout, work-related burnout, and client-related burnout. Personal burnout is measured with 6 items that assess general burnout and can be completed by anyone, regardless of occupational status. Work-related burnout is measured with 7 items that ask respondents to rate the degree of physical and psychological fatigue related to work. The client-related burnout subscale includes 6 items that measure fatigue and exhaustion related to caring for others^36^.

### Prevalence of burnout

All authors reported on the prevalence of burnout in their study population, using either proportions, means or medians. Prevalence of work-related burnout (as measured with the Copenhagen Burnout Inventory) was highest among Australian midwives, especially those working in non-caseload/continuity models (median scores of 48.4 and 46.4)^14,21^ and Western Canadian midwives (median score 46.4), and lowest among Norwegian midwives (mean score 19.1)^7^. Scores on the Maslach Burnout Inventory were lowest among midwives in Turkey (mean score on the emotional exhaustion (EE) subscale was 13.9) and the Netherlands (mean score 19.9)^29^, and highest among midwives in Senegal (80% scored in the moderate to high range on the EE subscale)^16^. Prevalence of burnout varied by model of care, with case loading midwives consistently reporting less burnout than midwives who work in other models^13,15,17,18,20,21^. Most of this evidence comes from Australia and New Zealand.

Studies utilizing burnout inventories were able to expand on their findings by describing the prevalence of burnout subdomains. Of those using the MBI, the ‘emotional exhaustion’ subscale emerged as the most frequently cited dimension of burnout, followed by ‘depersonalization’, and then ‘personal accomplishment’^11,31,32^. Respondents with a high score in emotional exhaustion and depersonalization, and a low score in personal accomplishment are considered severely burnt-out. In their sample of 56 Australian midwives, Mollart et al.^11^ found 60.7% to have moderate to high levels of emotional exhaustion, higher than in other studies from the US (41.8 %)^30^ and Iran (41.9 %)^31^ but lower than those reported by midwives in Senegal (80%)^37^. A similar pattern emerged amongst studies using the CBI: ‘personal burnout’ was the most prevalent dimension of burnout, followed closely by ‘work-related’ burnout, and then ‘client-related’ burnout in a distant third-place^3,7,19-21,27^. Stoll and Gallagher^3^ reported the moderate-to-severe burnout prevalence of these subscales amongst Canadian midwives as 74.9%, 42.5%, and 20.3%, respectively, compared to Fenwick et al.^20^ who reported these figures as 64.9%, 43.8%, and 10.4%, respectively, among Australian midwives.

### Factors associated with burnout

After reviewing the articles, 26 factors that were significantly associated with burnout were identified (Table 2). These included both organizational factors (e.g. insufficient organizational support /distress related to organizational structure /poor or stressful work environment /poor perceptions of practice environment, not enough time off, poor pay) as well as individual factors unique to the midwife (e.g. young age, less work experience, marital status).

The most commonly reported factors linked to burnout were: insufficient organizational support for profession; poor or stressful work environment^3,7,15,27,28,30,33^; working in non-case-load/non-continuity models of care (such as hospital shift work)^13,15,17,18,20,21^; less work experience in maternity care^11,19,27,29,30,34^; younger age^7,15,19,27,30,31^; high workload/not enough time off^3,15,23,27,30^; trauma/stress experienced at work^22,25,26,37^; interpersonal conflict with colleagues/low recognition of midwives^15,27,28,34^. For a full list of factors, see Tables 1 and 2.

### Recommendations

Most authors offered recommendations for improving working conditions for midwives and reducing the prevalence of burnout. There was a diversity of suggestions offered to reduce midwife burnout, many of which overlap with and appear to target factors identified in Table 2.

The most widely reported recommendations were: offering more work-related education, improving organizational support, and working in a caseload-model of midwifery practice. Other recommendations included: better support after traumatic events, education for midwives to learn ways of coping with occupational stress and inter-professional education or programs to reduce inter-professional bullying and conflict. Less frequently reported suggestions included: offering part-time work options or career development opportunities, and promoting exercise and physical activity, as a way to reduce stress. See Table 1 for a full description of recommendations.

## DISCUSSION

The purpose of this article was to present an up-to-date and comprehensive review of the existing literature on burnout in midwifery, with a particular focus on understanding the factors that are associated with burnout. In total, we included 27 peer-reviewed articles meeting our stated inclusion criteria. The findings of this scoping review lend some credence to previous calls of alarm, depicting a field fraught with high occupational burnout, and identifying several associated factors and recommendations to address it.

Despite the breadth and diversity of the literature across geography, measures, models of practice, and sample size and composition, our review noted that all included studies featured quantitative research methods, most often cross-sectional study designs. While these methodologies are certainly valuable for producing data that can be easily compared across groups and countries, they do little to answer the ‘why’ or ‘how’ questions that could shed light on the lived experience of midwives who are struggling with burnout. Without disparaging the value and significance of the existing research in broadening our understanding of burnout in midwifery, the addition of qualitative research studies would provide much needed insight into how midwives experience burnout, and more meaningfully involve midwives in identifying factors and possible solutions. This may be especially relevant when exploring sensitive topics, such as burnout and mental health. Cross-sectional designs, which allow researchers to explore associations between variables, prevent us from identifying causal and temporal effects between burnout and other factors. Longitudinal study designs would allow us to ascertain which factors cause burnout or which solutions or strategies alleviate it. Only one paper from the review utilized a longitudinal study design, a study in Senegal that measured job satisfaction in a cohort of 226 midwives over a three-month period, and then two years later examined the effect on burnout, intention to quit, and job turnover^16^.

While not the crux of our investigation, it quickly became apparent that, when reported, response rates for surveys tended to be low, often much less than 50%. A low response rate may be concerning as it might indicate a higher potential for sampling or non-response bias, should the included respondents not be representative of the midwifery population being studied. Given that most results were obtained via voluntary, self-reported questionnaires, it may be assumed that those experiencing burnout, or those more familiar with the topic, were more likely to complete and return the questionnaires. This would ultimately overestimate the prevalence of burnout in midwifery. Alternatively, it could also be theorized that those suffering the most from burnout were less likely to complete the questionnaires because of their ‘burnt-out’ state and as a result underestimate the true prevalence of burnout in the profession. Irrespective, future studies investigating burnout amongst midwives, especially those limited to self-reported surveys, should implement strategies to increase the response rate. A 2009 study investigating low response rates in postal surveys of healthcare professionals found that while response rates were not significantly different between healthcare professions, they were higher when reminders to complete the surveys were sent39.

### Burnout measures

We used different assessment tools to measure burnout. The most commonly used tools for measuring burnout were the MBI^35^ and CBI^36^. An important difference between these two scales is their theoretical underpinnings: the MBI describes burnout as a syndrome of depersonalization, reduced personal accomplishment, and emotional exhaustion as related to ‘people work’^35^, whereas the CBI describes burnout as ‘fatigue and exhaustion’ resulting in personal, work-related and client-related burnout^36^. While these differences may appear to be nuanced, they ultimately make it difficult to make direct comparisons between burnout studies. We recommend that future studies consider using the CBI in preference to the MBI for several reasons including: ‘depersonalization’ is perhaps better seen as a coping mechanism rather than a dimension of burnout, the MBI questions do not always adapt well to diverse cultures, and to reduce potential issues regarding distribution rights as some versions of the MBI are not in the public domain^35^.

### Burnout factors

In total, 26 factors were associated with burnout in the included literature and were reported in this scoping review. These factors could be broadly stratified into: 1) sociodemographic or lifestyle factors of the midwife (e.g. age, activity level, physical health, parental and relationship status); and 2) systemic and organization factors that affect the midwife (e.g. level of autonomy, inadequate facilities, low wage). The most widely supported factors for burnout included an approximately equal proportion of these two categories, suggesting that of the two there is no single domain that is disproportionately associated with burnout in the profession. ‘Less work experience in maternity care’ and ‘younger age’ are interrelated factors that emerged as the sociodemographic characteristics receiving support from the greatest number of articles. ‘Insufficient organizational support /poor or stressful work environment’, ‘practicing in non-caseload/non-continuity models of care’ (such as hospital shift work), and ‘high workload’, were the most prominent systemic and organizational-related factors found in the literature. Interventions for addressing burnout among midwives, therefore, may wish to consider avenues for reducing workload (e.g. hiring more midwives on staff, enabling case-load midwives to take fewer clients) – especially for midwives that are of young age or are early in their careers. Closely related to reducing work-load is remuneration of midwives. Higher pay enables midwives to take on fewer shifts or carry a smaller caseload.

The largest global survey of the midwifery work force to date revealed that midwives are deeply committed to their work, but experience many challenges. For example, midwives across low-, middle- and high-income countries reported loss of autonomy and power within the healthcare system as major barriers to providing high quality care. Disrespect from senior medical staff, low pay and lack of leadership opportunities were commonly reported and illustrate the uphill battle many midwives face^40^. Findings from this report and the current scoping review demonstrate the need to improve working conditions for midwives, so they can continue providing high quality care while also enjoying better work-life balance, emotional well-being and mental health^40^.

By their nature, factors characteristic of the midwife can be difficult and often impossible to change. Accordingly, hospital administrations and clinics should first look inward and address the policies and organizational factors that might contribute to burnout among midwives and other health professionals. A recent publication from the UK supports this point. Of the close to 2000 midwives who participated in an online survey, 83% reported personal burnout, and 67% work-related burnout^41^, placing UK midwives at the top of the list when compared to burnout scores from other high-income countries that utilized the CBI (Table 1). Perceived resource inadequacy was the strongest predictor of work-related burnout. Other factors associated with burnout included younger age (40 years or less), less work experience (<30 years), having a disability and reporting low levels of support from midwifery managers and low professional recognition^41^. These results are in line with the main findings of this scoping review.

Several additional contributing factors for burnout were identified in one of the studies. These included poor self-rated physical or mental health^27,38^, low levels of physical activity^11^, night shifts only compared to mixed shifts (day and night)^11^, practice location (home versus hospital)^13^, and coping style^13^. Future research should consider including these factors in their investigations, to better understand the role they may have in contributing to burnout.

Additionally, researchers examining burnout in midwifery may also want to consider factors known to contribute to burnout in physicians, including fears of litigation, the increasing reliance on technology, and the growing uncertainties regarding the future of medicine; none of these factors was explicitly explored in the included articles. Research on physician burnout also delves into how the nature of a physician’s tasks influences and aggravates their symptoms of burnout. Similar to the findings from this review, a study on Canadian physicians showed that 64% feel that their workload is excessive, and that 48% reported that their workload had increased in the past year^42^. One possible explanation for this perceived increase in workload has been the increase in mundane or clerical tasks, which have been shown to compromise a physician’s sense of job satisfaction^3^. Perhaps the same can be applied to midwives: clerical tasks and other responsibilities, not directly related to healthcare, may cause them to experience a high workload and contribute to burnout. Two of the included studies^37,38^ reported significant linkages between burnout and low task satisfaction. Further research on the impact of non-healthcare tasks have on the overall workload of midwives is warranted.

It is important to mention that the frequency of specific burnout factors across studies does not necessarily translate into how much significance (or ‘weight’) they should be assigned. A widely identified burnout factor may contribute only a small portion towards the prevalence or severity of burnout in midwifery (and vice versa for a seldom mentioned factor). This uncertainty on the weight each factor should be given is further compounded by the ambiguous or ‘broaden-compassing’ nature of many of the specified factors. For example, in the case of ‘high workload’ it is not always clear how or by what measure workload is being considered or interpreted by survey respondents. This area of uncertainty regarding how burnout factors interplay, specifically how to aggregate or separate factors, and to what degree individual factors contribute to burnout would certainly benefit from additional qualitative research.

Finally, this global scoping review included studies from many different high-resource countries and one low-resource country. The context for midwifery practice is very different across countries and affect the way midwives experience work and work-related burnout. It is of interest to note that several of the included studies were conducted as part of an international working group of midwives and researchers called WHELM – Work, Health and Emotional Lives of Midwives^14^. The WHELM group uses a standardized survey to collect data about burnout, occupational stress, intentions to leave, quality of life and other factors, from midwives in many high-resource countries, including Australia, New Zealand, the UK, Canada, Germany, and Scandinavia. In the future, data from midwives from different countries participating in the WHELM study might be pooled, to better understand similarities and differences in how burnout is experienced by midwives in different countries.

### Burnout recommendations

‘Offering more work-related education’ emerged as a commonly cited recommendation. However, the content and purpose of suggested additional education varied across the studies. Offering more work-related education included: further training on increasing autonomy and clinical decision making^19^, educational workshops on preventing and addressing work-related burnout^31^, and ongoing education and clinical mentorship, to increase clinical competency and build confidence^19,37^.

Offering midwives the option to practice a caseload model of midwifery care was found to be associated with reduced burnout. In a recent cross-sectional survey of 542 Australian midwives, a direct comparison between caseload and non-caseload midwifery revealed the latter group scored higher on all three CBI subscales of burnout (p<0.001)^17^. These results are supported by other studies demonstrating that midwives practicing caseload midwifery experience less burnout than those working in non-caseload models^13,15,18,20,21^. These findings present a clear and unanimous recommendation for policy makers and healthcare administrators seeking to address burnout through changes in practice models by supporting caseload models. Studies about caseload midwives can be informative in terms of anticipating factors linked to burnout among midwives who practice this model of care. For example, Stoll and Gallagher^3^ studied case-load midwives in Western Canada. Their recommendations to reduce burnout were based on open-ended comments from midwives about strategies to reduce burnout and increase job satisfaction. Recommendations fell into four general areas: more time off /better pay, more flexible practice structures /change in model of care, more respect from profession /more support from colleagues and more support with professional issues (such as help with obtaining hospital privileges or more support after critical incidents). Specific recommendations within these four areas included: part-time work options, support for sick days/vacation coverage, more pay per course of care and more pay for complex clients, salaried rather than fee-for-service payment schemes in rural and remote areas, more off-call career opportunities, and initiatives to reduce intra-professional bullying and inter-professional conflict^3^.

Similar to the concerns raised in the previous section, the level of support for a particular burnout-reduction strategy across the included literature may not necessarily be representative of its success or effectiveness in reducing burnout, should it be implemented. These inquiries would be best addressed with additional investigation, in particular from studies utilizing longitudinal designs evaluating the efficacy of these recommendations.

Burnout is a complex issue that requires complex interventions. Because it is an occupational phenomenon, the onus for change is placed on organizations, such as hospitals, professional organizations, health policy makers and regulatory bodies. Finally, this review uncovered some inconclusive findings. For example, two studies identified that being married is linked to burnout^24,38^ whereas two other studies identified being single or divorced as a risk factor^7,30^. Similarly, high work-load and long hours were linked to burnout in some studies^3,15,23,30^ but in another study midwives who worked more hours per week also reported higher scores on the personal accomplishment subscale of the MBI^29^. Future studies might shed light on how these factors relate to burnout, ideally using qualitative study designs. Such designs can also elicit detailed responses about the kind of partner support that buffers against burnout and how work autonomy relates to burnout.

### Limitations

This scoping review is not without its limitations. First, given the evolving nature of occupational burnout as a unique state distinct from other psycho-social constructs, the search terms used for this review may not have been fully inclusive of all the terminology used – presently and historically – to describe burnout. This may have resulted in certain relevant papers not being considered for inclusion. Our review was also limited by screening out articles that were not available in English, or those that were not accessible by database subscriptions held by the University of British Columbia. Further, there may be publication bias as a consequence of studies with significant findings being preferentially selected by journals for publication – and hence inclusion in this review. Finally, this scoping review did not include a quality assessment of articles.

## CONCLUSIONS

The importance of midwives as primary maternal-fetal health care providers is undeniable, underscoring the need to retain midwives in the profession. Unfortunately, many midwives experience burnout, which leads to attrition, and impairs their ability to provide high quality care. This scoping review examined the literature to better understand these elements, and coalesced 27 relevant articles to describe the prevalence of burnout, factors associated with burnout, as well as some recommendations to address this serious issue. In total, 26 factors associated with burnout were identified. Amongst recommendations, more work-related education, improved organizational support, increased personal resources, and caseload models of practice emerged as the most widely supported. Future investigations should consider adding to the largely absent body of qualitative research in this area, to better understand the ‘how’ and ‘why’ of these burnout factors and recommendations.
